# Genetic variation in the CYP2B6 Gene is related to circulating 2,2’,4,4’-tetrabromodiphenyl ether (BDE-47) concentrations: an observational population-based study

**DOI:** 10.1186/1476-069X-13-34

**Published:** 2014-05-08

**Authors:** Johanna Penell, Lars Lind, Tove Fall, Anne-Christine Syvänen, Tomas Axelsson, Per Lundmark, Andrew P Morris, Cecilia Lindgren, Anubha Mahajan, Samira Salihovic, Bert van Bavel, Erik Ingelsson, P Monica Lind

**Affiliations:** 1 Department of Medical Sciences, Occupational and Environmental Medicine, Uppsala University, Uppsala, Sweden; 2 Department of Medical Sciences, Cardiovascular Epidemiology, Uppsala University, Uppsala, Sweden; 3 Department of Medical Sciences, Molecular Epidemiology and Science for Life Laboratory, Uppsala University, Uppsala, Sweden; 4 Department of Medical Sciences, Molecular Medicine and Science for Life Laboratory, Uppsala University, Uppsala, Sweden; 5 Wellcome Trust Centre for Human Genetics, University of Oxford, Oxford, UK; 6 MTM Research Centre, School of Science and Technology, Örebro University, Örebro, Sweden

**Keywords:** 2,2’,4,4’-tetrabromodiphenyl ether, BDE-47, CYP, Elderly, Epidemiology, Gene, SNP

## Abstract

**Background:**

Since human *CYP2B6* has been identified as the major CYP enzyme involved in the metabolism of 2,2’,4,4’-tetrabromodiphenyl ether (BDE-47) and that human 2B6 is a highly polymorphic CYP, with known functional variants, we evaluated if circulating concentrations of a major brominated flame retardant, BDE-47, were related to genetic variation in the *CYP2B6* gene in a population sample.

**Methods:**

In the population-based Prospective Investigation of the Vasculature in Uppsala Seniors (PIVUS) study (men and women all aged 70), 25 single nucleotide polymorphisms (SNPs) in the *CYP2B6* gene were genotyped. Circulating concentrations of BDE-47 were analyzed by high-resolution gas chromatography coupled to high-resolution mass spectrometry (HRGC/ HRMS).

**Results:**

Several SNPs in the *CYP2B6* gene were associated with circulating concentrations of BDE-47 (*P* = 10^-4^ to 10^-9^). The investigated SNPs came primarily from two haplotypes, although the correlation between the haplotypes was rather high. Conditional analyses adjusting for the SNP with the strongest association with the exposure (rs2014141) did not provide evidence for independent signals.

**Conclusion:**

Circulating concentrations of BDE-47 were related to genetic variation in the *CYP2B6* gene in an elderly population.

## Background

Polybrominated diphenyl ethers (PBDEs) are flame-retardant chemicals that are commonly used in textiles such as clothes and furniture, electronic equipment, plastics and various inflammable materials to prevent fires [1,2]. The Stockholm Convention in 2009 listed penta- and octa-PBDE as new persistent organic pollutants and classified them as materials requiring international regulation [3]. The use of certain PBDEs has been restricted or banned in the European Union since 2004. However, due to their persistence in the environment these chemicals have become ubiquitous environmental pollutants and can be detected in air, soil and water. Despite the growing concern there is, in general, little information about the risks these chemicals pose to public health [4]. Concentrations of serum PBDEs has been associated with self-reported hand-to-mouth behaviors, including biting nails and licking fingers, and ownership of a large-screen TV [5]. PBDEs have a neurotoxic potential and higher concentrations of PBDE have been associated with various adverse outcomes in children such as impaired cognitive and behavior development [6]. Furthermore, associations between PBDEs (BDE 47, 99 and 100) in house dust and hormone levels in men have been reported [7]. Considering the severe adverse health effects reported in relation to PBDE concentrations, it is of outmost importance to increase the understanding of PBDE metabolism. 2,2’,4,4’-tetrabromodiphenyl ether (BDE-47) is the most commonly detected PBDE congener in human serum worldwide [8-13]. We have previously reported concentrations of BDE-47 in the majority of elderly men and women in a population-based sample [14]. We use this congener as a marker for PBDE exposure as it is frequently detected and included in a large number of biomonitoring studies and thus valid for comparison.

Enzymes produced from the cytochrome P450 (CYP) genes are primarily found in the liver and they are involved in the synthesis and metabolism of various molecules and chemicals within cells [15]. Apart from the role CYP enzymes play in the synthesis of steroid hormones, certain fats, and acids used to digest fats, they are also involved in metabolizing endogenous toxins and xenobiotics for example pharmaceuticals and environmental pollutants such as PBDEs [16].

The metabolism of brominated flame retardants has been extensively studied in the experimental setting. In human liver microsomes, CYP2B6 plays a key role in the biotransformation of many xenobiotics [17] and has been identified as the major CYP enzyme involved in the metabolism of BDE-47 [18,19]. Furthermore, in rat hepatoma cells and in zebrafish embryos, a BDE-47 mixture enhanced *CYP2B* gene expression [20]. Also, studies with recombinant CYPs support CYP2B6 as the major CYP in CYP-mediated metabolism of BDE-47 [19].

The activity of an enzyme and the gene expression may be modified by single nucleotide polymorphisms (SNPs) [17]. The *CYP2B6* sequence carries high variance [21,22] and up to today at least 29 variants have been described [23]. A majority of the variation in CYP450 activity relates to SNPs in the *CYP450* gene locus [21]. Certain SNPs in the region have a large impact on CYP activity [24] such as altering the composition of transcribed protein [21] and affect the interindividual differences in xenobiotic metabolism [25]. Several single nucleotide polymorphisms (SNPs) in the P450 system have been reported to influence the activity of different CYP enzymes that are important for metabolism of both endogenous, exogenous and xenobiotic compounds [21,25,26]. For example, a specific genotype in *CYP2B6* has been shown to affect the xenobiotic metabolism of an anti-HIV agent [27]. Also, effects of certain SNPs have been reported, for example rs2279345 was found to be associated with a higher clearance and a lower plasma concentration of methadone [28]. On the contrary, several SNP polymorphisms (including rs8192719) were underrepresented in the low-methadone group suggesting decreased CYP2B6 activity [29]. When 13 SNPs in *CYP2B6* (five of which are included in this study) were investigated regarding plasma efavirenz (a HIV-medication) concentrations, 3 SNPs (rs10403955 (not investigated in this study), rs2279345 and rs8192719) were found representative of the 11 SNPs associated with plasma efavirenz concentrations [30]. There is evidence on species differences in the metabolism of BDE-47 [16]. However, for humans, there is a lack of knowledge regarding the impact of SNPs in *CYP2B6* on BDE-47 metabolism and plasma concentrations in the real-life setting. Such information may provide clinically useful information on recommendations to reduce exposure to BDE-47 in certain susceptible individuals. This study investigated whether the experimental findings on the associations between SNPs in *CYP2B6* regarding BDE-47 concentrations could be repeated in an epidemiological cross-sectional observational study in humans. We used data from the Prospective Investigation of the Vasculature in Uppsala Seniors (PIVUS) study to study if SNPs in cytochrome P450 2B6 (*CYP2B6*) gene, encoding the only enzyme in the *CYP2B* subfamily that is expressed in the liver in humans, were related to circulating concentrations of BDE-47 in men and women by applying multivariable linear regression model techniques.

## Methods

### Study population

All men and women aged 70 and living in the community of Uppsala, Sweden at enrolment between April 2001 and June 2004 were eligible for the study. Two thousand and twenty five participants were randomly selected from the national register of residence and invited to the study. A total of 1016 subjects participated (participation rate 50.1%). The study was approved by the Ethics Committee of the University of Uppsala (diary number 2007/302) and all the participants gave their written informed consent.

All subjects were investigated in the morning after an over-night fast. No medication or smoking was allowed after midnight. The participants also answered a questionnaire about their medical history, regular medication, and smoking and exercise habits. Approximately 10% of the cohort reported a history of coronary heart disease, 4% reported stroke and 9% reported diabetes mellitus. Almost half the cohort reported some sort of cardiovascular medication (45%), with antihypertensive medication being the most prevalent (32%). Fifteen percent reported use of statins, while oral antiglycemic drugs and insulin use were reported in 6 and 2%, respectively. Eleven percent were current smokers, see reference [31] for details. Blood samples were taken through an arterial cannula inserted in the brachial artery [31].

### BDE-47 analysis

BDE-47 concentrations were measured in stored plasma samples collected at the baseline investigation using a high-resolution gas chromatograph (6890 N, Agilent Technologies, Atlanta, GA, USA) coupled to a high-resolution mass spectrometer (Micromass Autospec Ultima,Waters, Milford, MA, USA) (HRGC/HRMS) based on the method by Sandau and co-workers [32] with some modifications. A more detailed description of the analysis in this sample has previously been presented [33].

In 255 (28%) of the persons included in the analyses, BDE-47 was below the detection limit (9.2 pg/ml). In those subjects the value was set to the detection limit divided by the squared root of two, which is a commonly used method to impute censored data [34]. This has been recommended when relatively few data are below the detection limit or where data are not highly skewed [35]. Therefore, this procedure has been widely used because of its simplicity and ease of operation and produce low bias and moderate precision using environmental data [36]. Lipid-normalized values (ng/g lipid) for BDE-47 were used in the analysis. The total amount of lipids in each plasma sample was obtained by using an established summation formula based on serum cholesterol and serum triglyceride concentrations [37]. In the next step, using the wet-weight concentration of the BDE-47 as nominator and the amount of lipids as the denominator, lipid-normalized BDE-47 values were obtained.

### Genotyping

A total of 25 SNP in the P450 enzyme *CYP2B6* gene were genotyped using the Illumina OmniExpress chip at Wellcome Trust Sanger Institute, Oxford, UK (20 SNPs) and a custom Illumina Golden Gate assay at the SNP&SEQ Technology Platform at Uppsala University, Uppsala, Sweden (http://www.genotyping.se) (5 SNPs). Sample exclusion criteria included: 1) genotype call rate <95%; 2) heterozygosity >3 standard deviations (SDs); 3) gender discordance; 4) duplicated samples; and 5) no identity-by-descent match. SNP exclusion criteria included: 1) monomorphic SNPs; 2) Hardy-Weinberg equilibrium (HWE) p-value < 1×10-6 for the GWAS selected SNPs and *P* < 0.05 for the directly genotyped SNPs; 3) genotype call rate <0.99 for SNPs with minor allele frequency (MAF) <5%, or <0.95 for SNPs with MAF ≥ 5%. One SNP (rs1272165) failed to meet SNP exclusion criteria 3, and hence was excluded from further analyses.

Using the European 1000 Genomes genotypes as reference (population codes: CEU, FIN, GBR, IBS, TSI), the gene region for *CYP2B6*, length 41.9 kb, contained 128 SNPs with MAF > 5%. The SNPs genotyped in this study could tag 110 of these at r^2 > 0.8. All SNPs genotyped in the study were present in the 1000 Genomes data.

### Statistics

BDE-47 concentrations were transformed to the natural logarithm scale prior to analysis to account for the non-normal distribution. Relationships between the *CYP2B6* SNPs and BDE-47 concentrations were evaluated by linear regression analysis with BDE-47 as dependent linear variable adjusting for gender and the principal components 1 and 2 (derived from EIGENSTRAT based on GWAS data). Each SNP was coded as 0, 1 or 2 alleles of the minor allele and evaluated in a separate multivariable model assuming additive allele effect. The p-values were adjusted to account for multiple testing (see below). Principal components were included to account for any possible population stratification in the study population that may bias the relationships under study. To investigate if there were several independent signals, the analyses were also performed for the remaining SNPs when adjusting for the lead (i.e. lowest p-value) SNP in addition to gender and the principal components 1 and 2 (i.e. conditional analysis). To investigate if associations between BDE-47 and SNP were stable regardless if the BDE-47 concentrations were above or below the detectible limit, logistic regression analysis with a binary variable for non-detectable/detectable BDE-47 concentrations as dependent variables and the SNPs, gender and the principal components 1 and 2 as independent variables was performed. Since 24 tests were performed in the main linear and logistic regression analyses, we adjusted the critical p-value accordingly using the Bonferroni correction (p-value = 0.05/24 = 0.0021 for statistical significance). The use of this strict threshold, not taking into account the high correlations of SNPs within the same LD blocks which would warrant a more liberal threshold, was motivated by the lack of suitable replication samples (see further in the Discussion section).

## Results

The number of study subjects included in the analyses was 924 of which 462 were women (50%). The mean value for the lipid-normalized BDE-47 was 4.3 (SD 20.1, range 0.6-476) ng/g lipid. The median value was 2.0 (1.2-3.0 for 25^th^ and 75^th^ percentile) ng/g lipid.

**Figure 1:**
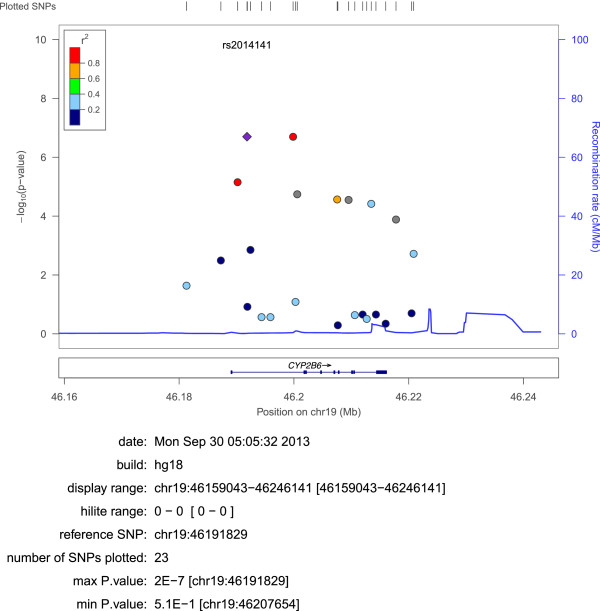
**Association signal plot for SNPs mapping to *CYP2B6.*** Each point represents a SNP in the association analysis, plotted with their p-value (on a -log10 scale) as a function of genomic position (NCBI Build 36). The lead SNP (with maximal association signal) is represented by the purple symbol. The shape of the plotting symbol corresponds to the annotation of the SNP: square for synonymous or UTR; and circle for intronic or non-coding. The colour coding of all other SNPs indicates LD with the lead SNP (estimated by CEU r2 from Phase II HapMap): red r2 ≥ 0.8; gold 0.6 ≤ r2 < 0.8; green 0.4 ≤ r2 < 0.6; cyan 0.2 ≤ r2 < 0.4; blue r2 < 0.2; grey r2 unknown. Recombination rates are estimated from Phase II HapMap and gene annotations are taken from the University of California Santa Cruz genome browser.

**Figure 2:**
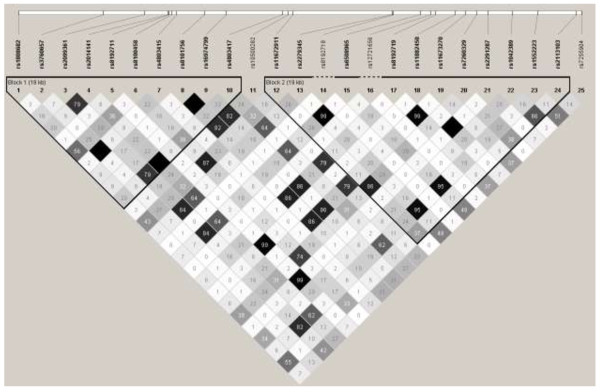
**Display over the haplotype structure of the *CYP2B6*-loci, based on PIVUS genotype data.** Darker color corresponds to higher r^2^ (i.e. correlation) between single nucleotide polymorphisms (SNPs).

The associations between 24 SNPs in the *CYP2B6* gene and circulating concentrations of lipid-normalized BDE-47 are presented in Table 1. For example, in Table 1 the beta coefficient for rs2014141 versus lipid-normalized ln-transformed BDE-47 was -0.21. This should be interpreted as that the per-allele effect of the SNP will correspond to a 0.21 decrease in lnBDE-47 concentrations which corresponds to e^-0.21^ = 19% decrease in total serum BDE-47 concentrations. Figure 1 shows the association signal plot for SNPs mapping to *CYP2B6* with BDE-47. The haplotype structure of the *CYP2B6* SNPs based on the PIVUS data is shown in Figure 2.

**Table 1:** **Association between SNPs in the *CYP2B6 *** **gene and circulating lipid-normalized levels of BDE-47**

**SNP**^**a**^	**position**	**Minor allele/major allele**	**MAF**^**b**^	**n**_**obs**_**in the analysis**	**Beta (95% CI) on natural logaritm scale**	**P-value (Bonferroni-corrected p-value for significance = 0.0021)**
rs1808682^c^	41489448	A/G	0.23	920	0.11 (0.026, 0.19)	9.5*10^-3^
rs3760657	41495433	G/A	0.12	924	0.16 (0.058, 0.27)	2.5*10^-3^
rs2099361	41498348	C/A	0.36	923	-0.18 (-0.25, -0.11)	2.8*10^-7^
rs2014141	41499989	A/G	0.41	924	-0.21 (-0.28, -0.14)	4.9*10^-9^
rs8192711	41500057	A/G	0.05	923	-0.12 (-0.27, 0.031)	0.12
rs8100458	41500213	C/T	0.35	924	0.17 (0.10, 0.24)	3.4*10^-6^
rs4803415	41500593	T/C	0.10	886	0.19 (0.08, 0.30)	6.9*10^-4^
rs8101756	41502522	C/T	0.24	923	0.062 (-0.018, 0.14)	0.13
rs16974799	41504077	T/C	0.24	924	0.062 (-0.018, 0.14)	0.13
rs4803417	41508020	C/A	0.41	924	-0.21 (-0.28, -0.14)	4.9*10^-9^
rs10500282	41508442	C/T	0.26	923	0.087 (0.009, 0.16)	0.028
rs11672911	41508744	A/G	0.32	924	0.17 (0.10, 0.25)	5.6*10^-6^
rs2279345	41515702	T/C	0.39	924	-0.17 (-0.24, -0.10)	9.6*10^-7^
rs8192718	41515814	A/G	0.02	924	-0.099 (-0.39, 0.19)	0.50
rs6508965	41517688	T/C	0.39	924	-0.17 (-0.24, -0.10)	1.1*10^-6^
rs8192719	41518773	T/C	0.24	924	0.066 (-0.012, 0.14)	0.10
rs11882450	41520142	G/A	0.06	924	-0.095 (-0.24, 0.052)	0.20
rs11673270	41520844	C/A	0.24	924	0.060 (-0.019, 0.14)	0.14
rs7260329	41521638	A/G	0.32	924	0.17 (0.093, 0.24)	9. 3*10^-6^
rs2291287	41522451	G/A	0.06	924	-0.095 (-0.24, 0.052)	0.20
rs1042389^c^	41524153	G/A	0.21	923	0.040 (-0.041, 0.12)	0.33
rs1552223	41525952	G/A	0.39	924	-0.16 (-0.23, -0.092)	5.0*10^-6^
rs2113103^c^	41528667	A/G	0.15	924	0.076 (-0.018, 0.17)	0.11
rs7255904^c^	41529020	A/G	0.45	921	0.13 (0.059, 0.19)	2.6*10^-4^

^a^SNP = single nucleotide polymorphisms.

^b^MAF = Minor allele frequency.

^c^SNPs genotyped in Uppsala, others in Oxford.

The position data originates from the UCSC Genome Browser on Human February 2009 (GRCh37/hg19) and allele data originates from the genotyped SNPs included in the study. All SNPs are located on chromosome 19.

Circulating BDE-47 concentrations were significantly associated with several of the investigated *CYP2B6* variants, even after taking multiple testing into account. The associated SNPs came primarily from two haplotypes (Figure 2; all p = 2.6*10^-4^ to 4.9*10^-9^). The two top SNPs rs2014141 and rs4803417 were in complete LD. The median values for the lipid-normalized BDE-47 for 0, 1 and 2 copies of the minor allele for rs2014141 were 4.3 (1.8-3.7 for 25^th^ and 75^th^ percentile), 4.8 (1.2-2.9 for 25^th^ and 75^th^ percentile) and 3.0 (1.1-2.3 for 25^th^ and 75^th^ percentile) ng/g lipid, respectively. Thirty five percent of the subjects had 0 copies of the minor allele whereas 17 percent had 2 copies of this allele. In addition, one SNP with MAF of 0.10 showed significant association with the outcome (rs4803415, p = 6.9*10^-4^). While close relationships were seen between the SNPs within the two haplotypes as expected, high LD (R^2^ > 0.90) was also observed between SNPs across the two haplotypes (Figure 2).

Conditional analyses were performed by adding one of the top SNPs (i.e. SNPs with lowest p-value from the main analyses, see Table 1), rs2014141, to the models of the other SNPs to investigate independent signals; none of the other 22 SNPs showed significant association with the BDE-47 concentrations.

**Figure 3:**
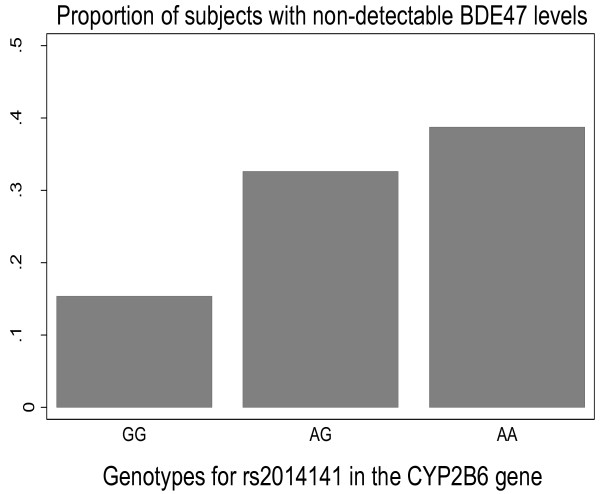
**Proportion of subjects with non-detectable BDE-47 levels according to the rs2014141 genotype in the *CYP2B6 *gene.** N_AA allele_ = 163, N_AG allele_ = 457, N_GG allele_ = 329.

Since 255 subjects showed BDE-47 concentrations below the limit of detection, we additionally performed logistic regression analysis with a binary variable for non-detectable/detectable BDE-47 concentrations as dependent variables and the SNPs, gender and the principal components 1 and 2 as independent variables in separate models for each SNP. That approach resulted in similar results as in the linear models described above, with highly significant associations between low BDE-47 concentrations and the genotypes with MAF in the 0.36-0.45 range (all p = 10^-4 to^ 10^-6^)(data not shown). Figure 3 shows the relationship between the proportion of non-detectable concentrations in the sample and genotypes for one of the top SNPs (rs2014141).

## Discussion

The present study, including a homogenous population of 70-year-old Caucasian men and women showed that genetic variation in the *CYP2B6* gene was related to circulating concentrations of the brominated flame retardant BDE-47. This finding is in line with previous experimental findings that BDE-47 was mainly bio transformed by CYP2B enzyme in human microsomes and could induce CYP2B enzymes in rats, respectively [18,20]. It has also been shown that BDE-47 induces CYP2B6 genes in mice and human hepatocytes, although at higher BDE-47 concentrations than generally found in humans [38,39]. There is yet no evidence that BDE-47 can induce CYP genes at the concentrations found in the general human population. However, if BDE-47 can induce CYP2B6 enzymes also at lower concentration, this will have implications on human response to numerous therapeutic drugs [39]. There is also evidence that other xenobiotics may induce or modulate *CYP2B6* in humans. For example the antiretroviral drug rilpivirine and a malaria drug were seen to inhibit CYP2B6 in vitro [40,41] as well as the anti-platelet drug ticlopidine in vivo [42]. Furthermore, carnosic acid which has been suggested as a potential treatment for obesity and nonalcoholic fatty liver disease has showed increased CYP2B6 enzyme activity thus indicating a potential drug interaction with carnosic acid [43]. In the present study, the strongest associations were seen between BDE-47 concentrations and the more common variants, which are also the most well-powered relationships to find. Additional conditional analysis adjusting for one of the top SNPs did not provide evidence of multiple signals. This implies that rs2014141 and rs4803417 might lie closest to the causal variant. Allelic heterogeneity (i.e. several causal mutations on different alleles/positions within a locus) cannot, however, be excluded due to the limited sample size and hence the low power to detect low frequency signals. Since we are not aware of any other population-based study with BDE measurements in a fairly large cohort in which also genetic analysis in CYP genes has been performed, we cannot replicate the findings in an independent cohort. Hence, we acknowledge the lack of replication as an important limitation, but believe that the study still is valuable due the high *a priori* probability for involvement of CYPs in the metabolism of brominated flame retardants. Also, the p-values well below the Bonferroni corrected p-values for statistical significance support the likelihood of the results not being chance findings.

Previously many studies have addressed the effect of variability in CYP2B6 on various xenobiotics [22,25,28,44]. Some of these studies include specific SNPs also investigated in this study. For example rs2279345 was associated with lower concentrations of methadone [28] and efavirenz plasma concentration [45] which is the same direction of the highly significant effect that is reported in this study for the intron variant rs2279345 on BDE-47 concentrations. Also rs8192719 has been reported to be related to methadone although in the opposite direction by lower CYP2B6 activity [29]. We did not see any association between this SNP and BDE-47 concentrations. Also rs1042389 has been investigated and found not be associated with response to two treatments of HIV infection [46] which mimics the no-existing relationship between this SNP and BDE-47 concentrations seen in this study. In addition, we compared the two top SNPs rs2014141 and rs4803417 identified in this study with previously known variants using HapMap release 22 and population panel CEU (http://www.broadinstitute.org/mpg/snap/ldsearch.php). This revealed that rs2014141 has perfect LD with rs6508964 and rs10418990 (r^2^ = 1.00), and high LD with rs2099361 and rs3889806 (both r^2^ = 0.87). For rs4803417, the correlation pattern was similar with high LD with SNPs rs6508964 and rs10418990 (r^2^ = 0.93), and with rs2099361 and rs3889806 (both r^2^ = 0.81). The so far most commonly studied *CYP2B6* SNP rs3745274 [30], which is a missense variant, where polymorphism is associated with for example lower hepatic expression and enzymatic activity for CYP2B6 [47], higher efavirenz exposure [48], plasma propofol concentrations [49] and increased breast cancer risk [50] was in low LD with the two top SNPs in this study (r^2^ = 0.29). Similarly, two other well-studied missense variants SNPs rs3211371 and rs2279343 associated with altered metabolism of various substrates [51] was also in low LD with the two top SNPs in this study (r^2^ = 0.29). Many other CYP2B6 SNPs have been reported to be associated with various phenotypes, however, none were in high LD with our two top SNPs. For example, neither SNPs related to nicotine metabolism (rs8109525, r^2^ = 0.28) [51] or to altered expression of the CYP2B6 enzymes (e.g. the missense variants rs8192709 and rs3211371, r^2^ < 0.2 ) [22], nor marker SNPs for plasma efavirenz concentrations (rs10403955 and rs8192719, r^2^ = 0.34 and 0.31, respectively ) [30] were in LD (r^2^ < 0.2) with rs2014141 and rs4803417.

The clinical implications of genotype polymorphism affecting BDE-47 concentrations are yet to be fully understood. We interpreted the findings in this study so that genotype may be a predictor of BDE-47 metabolism by affecting the BDE-47 concentrations. The suggested causal pathway from genotype to BDE-47 concentrations implies that genetic variability in the *CYP2B6* gene may lead to interindividual variability in the concentrations of BDE-47. This could have clinical relevance in subgroups with genotypes associated with higher PBDE concentrations or for individuals where PBDE concentrations should be kept to a minimum. For example, this would be of interest to pregnant women and parents bearing in mind the neurotoxicological, behavior and hormone disruptive effects that have been reported to be associated with PBDE exposure [6,7]. Taking advantage of genotyping has previously been suggested for example regarding selecting anaesthetics for optimal anesthesia that reduce risk of side effects as well as undesirable actions [52]. Analogously, as SNPs have been shown to affect the pharmacokinetics and response to a HIV-treatment drug [44] we suggest that detailed knowledge on SNP may be clinically relevant also regarding PBDE concentrations and metabolism.

Cytochrome P450 enzymes account for the majority of enzymes involved in drug metabolism. Common variations (polymorphisms) in cytochrome P450 genes can affect the function of the enzymes e.g. breakdown of medications. Indeed, several single nucleotide polymorphisms (SNPs) in the P450 system have been discovered to influence the activity of different CYP enzymes. Furthermore, a recent review on variations of cytochrome P450 in different ethnic populations suggests that ethnical differences in metabolic phenotype can be explained by differences in the distributions of SNPs [21]. Important substrates for *CYP2B6* include nicotine and the anti-depressant and smoking cessation aid bupropion [53,54]. As both BDE-47 concentrations [4] and ethnic composition of the population varies across the world, assessment of the effect of BDE-47 on *CYP2B6* on a wide range of exposure concentrations and different populations is relevant even though significant variation of CYP isoform activity may also occur within subgroups of a specific ethnic group [21]. In addition to being abundant in the liver, CYP2B6 is present in most regions of the brain [55]. This could be the reason why PBDE exposure has been associated with neurotoxicity and neurological diseases and could also reflect variability between individuals in response to centrally acting drugs.

The two haplotypes seen in this study agrees somewhat with earlier findings in that rs8100458 and rs2279345 were located in different haplotypes [28].

Ideally, the role of CYP induction on the metabolism of a xenobiotic, such as BDE-47, should be studied by distributing a fixed dose of the compound at a certain time point and thereafter evaluate the concentrations of the compounds in a standardized fashion. This is obviously not the case in the present observational epidemiologic study where there is no information on amount and timing of the BDE-47 exposure. However, by studying this relationship in an epidemiologic study including men and women, we may observe a relationship that more corresponds to real life settings. Furthermore, by including persons of the same age at a certain rather small time interval, we reduce variability due to age and time differences in exposure. Indeed, the major strength of the study is the rather large number of subjects with measurements of BDE-47 at the same age, which would increase the likelihood of establishing true relationships. A limitation of this study is that approximately one fourth of the sample had circulating concentrations of BDE-47 below the detection level. However, the additional analysis comparing observations with non-detectable and detectable concentrations of BDE-47 yielded similar results as the main findings indicating robustness of the study findings.

Future research on effects of BDE-47 exposure on humans may include proteome changes or differential protein expressions to determine if the molecular machinery could be adjusted to cope with e.g. BDE-47 exposure.

## Conclusions

Circulating concentrations of BDE-47 were related to genetic variation in the *CYP2B6* gene in an elderly population. Future research may investigate the protein expression pattern related to BDE exposure and determine if genetic adaptation to improve the resistance to BDE may occur in human populations as a response to this exposure.

## Abbreviations

BDE-47: 2,2’,4,4’-tetrabromodiphenyl ether; PIVUS: Prospective Investigation of the Vasculature in Uppsala Seniors; HRGC/HRMS: High-resolution gas chromatography/high-resolution mass spectrometry; PBDEs: Poly brominated diphenyl ethers; SNPs: Single nucleotide polymorphisms; WHO: World Health Organization; HWE: Hardy-Weinberg equilibrium; MAF: Minor allele frequency; CI: Confidence interval.

## Competing interests

The authors have no competing financial interests.

## Authors’ contributions

PML and LL conceived the project. EI contributed to the design of the study and the critical revision of the manuscript for important intellectual content. LL is the principal investigator of the Prospective Investigation of the Vasculature in Uppsala Seniors (PIVUS) study and performed the initial data analysis. PML and LL wrote the initial draft of the manuscript and contributed to the critical revision of the manuscript for important intellectual content. JP performed data analysis, had full access to all the data in the study and takes responsibility for the integrity of the data and the accuracy of the data analysis, and contributed to the critical revision of the manuscript for important intellectual content. S.S. and B.v.B. performed POPs laboratory analyses and contributed to the critical revision of the manuscript for important intellectual content. TF and APM carried out the association signal plot and the haplotype signal plot and contributed to the critical revision of the manuscript for important intellectual content. APM, CL, AM, AS, TA and PL carried out the genotyping and contributed to the critical revision of the manuscript for important intellectual content. All authors read and approved the final manuscript.
